# *cis*-Bis(2,2′-bipyridine-κ^2^ *N*,*N*′)dichloridocobalt(II) trihydrate

**DOI:** 10.1107/S1600536811009251

**Published:** 2011-03-19

**Authors:** K. Arun Kumar, M. Amuthaselvi, A. Dayalan

**Affiliations:** a Department of Chemistry, Loyola College (Autonomous), Chennai 600 034, India

## Abstract

In the title complex, [CoCl_2_(C_10_H_8_N_2_)_2_]·3H_2_O, the Co(II) ion is situated on a twofold rotation axis and exhibits a slightly distorted octa­hedral geometry and is chelated by four N atoms of the two bidentate 2,2′-bipyridine ligands and two Cl^−^ ions. The crystal packing is stabilized by hydrogen bonding formed between chloride ions and adjacent water mol­ecules. One of the two independent water molecules in the asymmetric unit is disordered over two sets of sites, each on a twofold rotation axis, in a 0.734 (17):0.269 (17) ratio.

## Related literature

For the anti­bacterial activity of similar complexes, see: Senthilkumar & Arunachalam (2008 ▶). For similar complexes applied in the immunoassay of carcinoma anti­gen-125, see: Shihong *et al.* (2009 ▶). For the application of similar complexes as biosensors, see: Ying *et al.* (2006 ▶).
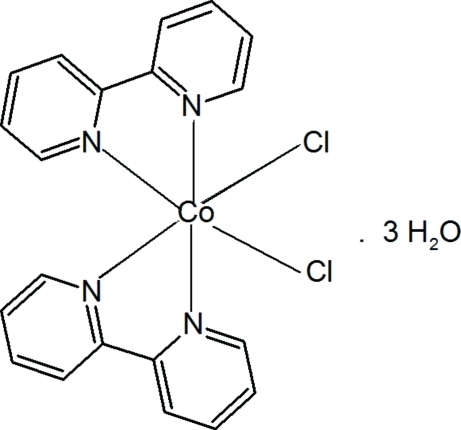


## Experimental

### 

#### Crystal data

[CoCl_2_(C_10_H_8_N_2_)_2_]·3H_2_O
*M* *_r_* = 496.25
Monoclinic, 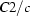

*a* = 18.3644 (8) Å
*b* = 13.1902 (8) Å
*c* = 10.8854 (6) Å
β = 120.030 (4)°
*V* = 2282.8 (2) Å^3^
*Z* = 4
Mo *K*α radiation
μ = 1.01 mm^−1^
*T* = 293 K
0.30 × 0.20 × 0.20 mm

#### Data collection

Bruker Kappa APEXII CCD diffractometer
Absorption correction: multi-scan (*SADABS*; Bruker, 2004 ▶) *T*_min_ = 0.721, *T*_max_ = 0.823
18022 measured reflections
2123 independent reflections
1731 reflections with *I* > 2σ(*I*)
*R*_int_ = 0.051

#### Refinement

*R*[*F* ^2^ > 2σ(*F* ^2^)] = 0.045
*wR*(*F* ^2^) = 0.143
*S* = 1.19
2123 reflections
152 parameters
4 restraints
H atoms treated by a mixture of independent and constrained refinement
Δρ_max_ = 0.67 e Å^−3^
Δρ_min_ = −0.65 e Å^−3^

### 

Data collection: *APEX2* (Bruker, 2004 ▶); cell refinement: *APEX2* and *SAINT* (Bruker, 2004 ▶); data reduction: *SAINT* and *XPREP* (Bruker, 2004 ▶); program(s) used to solve structure: *SIR92* (Altomare *et al.*, 1993 ▶); program(s) used to refine structure: *SHELXL97* (Sheldrick, 2008 ▶); molecular graphics: *ORTEP-3* (Farrugia, 1997 ▶) and *Mercury* (Bruno *et al.*, 2002 ▶); software used to prepare material for publication: *PLATON* (Spek, 2009 ▶).

## Supplementary Material

Crystal structure: contains datablocks I, global. DOI: 10.1107/S1600536811009251/bq2282sup1.cif

Structure factors: contains datablocks I. DOI: 10.1107/S1600536811009251/bq2282Isup2.hkl

Additional supplementary materials:  crystallographic information; 3D view; checkCIF report

## Figures and Tables

**Table 1:** Hydrogen-bond geometry (Å, °)

*D*—H⋯*A*	*D*—H	H⋯*A*	*D*⋯*A*	*D*—H⋯*A*
O1—H1*A*⋯Cl1	0.86 (6)	2.43 (5)	3.250 (4)	160 (5)
O1—H1*B*⋯Cl1^i^	0.85 (3)	2.37 (4)	3.218 (4)	172 (4)

Symmetry code: (i) 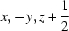.

**Fig. 1.**
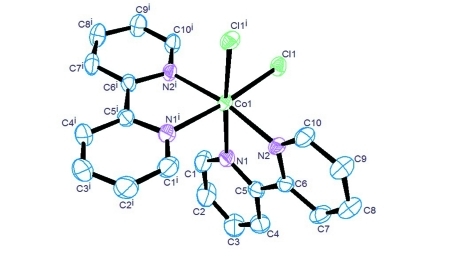
ORTEP of [Co(bpy)2(Cl)2].3H2O drawn with 50% displacement ellipsoid level. Water molecules have been omitted for clarity.

**Fig. 2.**
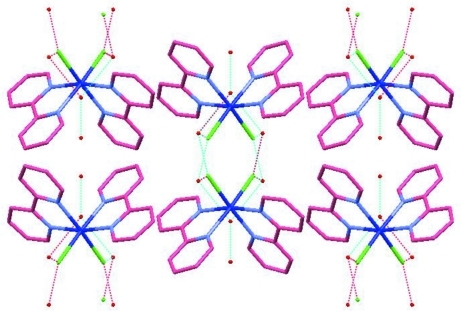
The crystal packing of [Co(bpy)2(Cl)2].3H2O viewed along the c axis.

## supplementary crystallographic information

### Comment

2,2-Bipyridine and 1,10-phenanthroline have been extensively used to form different complexes with transition metal ions in their various oxidation states. Tris(2,2'-bipyridine)cobalt(III) complexed with bovine serum albumin has been reported as biosensors (Ying *et al.*, 2006). Bipyridine cobalt complexes were found to have considerable antibacterial activities (Senthilkumar & Arunachalam, 2008). The use of tris(bipyridine)cobalt(II) for immunoassay of carcinoma antigen-125 has been reported recently (Shihong *et al.*, 2009).

The cobalt(II) ion has site symmetry 2 and assumes octahedral geometry with two symmetry related 2,2'- bipyridine ligands and two chloride ions. Cobalt(II) is linked to two 2,2'- bipyridine bidentate ligands *via* four nitrogen atoms and two chloride ions. The two 2,2'- bipyridine ligands are in *cis* position with mutually perpendicular to each other. The hydrated water molecules in the crystal packing helps the stabilization of crystal packing by forming hydrogen bonding between adjacent chloride ions.

### Experimental

The complex was prepared by adding a solution of 2,2'- bipyridine (0.01 mole) in 60 ml of acetone, to a solution of cobalt(II) chloride (0.005 mole) in 60 ml of acetone. The resulting solution was stirred for two hours, filtered and dried over vacuum desiccator to get red colour complex (yield 90%). The dark red colored crystals, suitable for x-ray analysis, were obtained by slow evaporation from alcoholic solution.

### Refinement

There is 1.5 water molecules per asymmetric unit. The H atoms of water oxygen O1 could be located in difference Fourier map. These H atoms were restrained to be at a distance of 0.85 Å from O1. The inter hydrogen distance was restrained to be 1.388 Å so as to retain the tetrahedral H—O—H angle. The other half molecule was disordered in two positions (O2 and O3). Their occupancies were refined initially as free variables and later the sum of the occupancies restrained as 0.5. The H atoms of disordered water molecules could not be located. The aromatic H atoms were constrained as riding atoms with d(C—H) = 0.93 Å and *U*_iso_(H) = 1.2*U*_equ_(C).

### Crystal data

**Table:**

[CoCl_2_(C_10_H_8_N_2_)_2_]·3H_2_O	*F*(000) = 1020
*M**_r_* = 496.25	*D*_x_ = 1.444 Mg m^−^^3^
Monoclinic, *C*2/*c*	Mo *K*α radiation, λ = 0.71073 Å
Hall symbol: -C2yc	Cell parameters from 6958 reflections
*a* = 18.3644 (8) Å	θ = 2.4–25.4°
*b* = 13.1902 (8) Å	µ = 1.01 mm^−^^1^
*c* = 10.8854 (6) Å	*T* = 293 K
β = 120.030 (4)°	Block, red
*V* = 2282.8 (2) Å^3^	0.30 × 0.20 × 0.20 mm
*Z* = 4	

### Data collection

**Table:**

Bruker Kappa APEXII CCD diffractometer	2123 independent reflections
Radiation source: fine-focus sealed tube	1731 reflections with *I* > 2σ(*I*)
graphite	*R*_int_ = 0.051
ω and φ scans	θ_max_ = 25.6°, θ_min_ = 2.0°
Absorption correction: multi-scan (*SADABS*; Bruker, 2004)	*h* = −22→19
*T*_min_ = 0.721, *T*_max_ = 0.823	*k* = −15→15
18022 measured reflections	*l* = −13→13

### Refinement

**Table:**

Refinement on *F*^2^	Secondary atom site location: difference Fourier map
Least-squares matrix: full	Hydrogen site location: inferred from neighbouring sites
*R*[*F*^2^ > 2σ(*F*^2^)] = 0.045	H atoms treated by a mixture of independent and constrained refinement
*wR*(*F*^2^) = 0.143	*w* = 1/[σ^2^(*F*_o_^2^) + (0.0754*P*)^2^ + 3.251*P*] where *P* = (*F*_o_^2^ + 2*F*_c_^2^)/3
*S* = 1.19	(Δ/σ)_max_ < 0.001
2123 reflections	Δρ_max_ = 0.67 e Å^−^^3^
152 parameters	Δρ_min_ = −0.64 e Å^−^^3^
4 restraints	Extinction correction: *SHELXL97* (Sheldrick, 1997), Fc^*^=kFc[1+0.001xFc^2^λ^3^/sin(2θ)]^-1/4^
Primary atom site location: structure-invariant direct methods	Extinction coefficient: 0.0015 (5)

### Special details

**Table:**

Geometry. All e.s.d.'s (except the e.s.d. in the dihedral angle between two l.s. planes) are estimated using the full covariance matrix. The cell e.s.d.'s are taken into account individually in the estimation of e.s.d.'s in distances, angles and torsion angles; correlations between e.s.d.'s in cell parameters are only used when they are defined by crystal symmetry. An approximate (isotropic) treatment of cell e.s.d.'s is used for estimating e.s.d.'s involving l.s. planes.
Refinement. Refinement of *F*^2^ against ALL reflections. The weighted *R*-factor *wR* and goodness of fit *S* are based on *F*^2^, conventional *R*-factors *R* are based on *F*, with *F* set to zero for negative *F*^2^. The threshold expression of *F*^2^ > σ(*F*^2^) is used only for calculating *R*-factors(gt) *etc*. and is not relevant to the choice of reflections for refinement. *R*-factors based on *F*^2^ are statistically about twice as large as those based on *F*, and *R*- factors based on ALL data will be even larger.

### Fractional atomic coordinates and isotropic or equivalent isotropic displacement parameters (Å^2^)

**Table:**

	*x*	*y*	*z*	*U*_iso_*/*U*_eq_	Occ. (<1)
C1	0.9376 (3)	0.3673 (3)	0.5122 (5)	0.0632 (11)	
H1	0.9858	0.3506	0.5089	0.076*	
C2	0.8848 (3)	0.4410 (4)	0.4210 (6)	0.0774 (14)	
H2	0.8973	0.4740	0.3583	0.093*	
C3	0.8134 (3)	0.4643 (4)	0.4254 (6)	0.0772 (14)	
H3	0.7768	0.5140	0.3658	0.093*	
C4	0.7964 (3)	0.4142 (3)	0.5176 (5)	0.0610 (11)	
H4	0.7476	0.4288	0.5202	0.073*	
C5	0.8517 (2)	0.3418 (2)	0.6070 (4)	0.0428 (8)	
C6	0.8377 (2)	0.2843 (2)	0.7097 (3)	0.0394 (8)	
C7	0.7668 (2)	0.2959 (3)	0.7214 (4)	0.0518 (9)	
H7	0.7247	0.3408	0.6623	0.062*	
C8	0.7588 (2)	0.2405 (4)	0.8212 (5)	0.0599 (10)	
H8	0.7120	0.2486	0.8319	0.072*	
C9	0.8211 (2)	0.1730 (3)	0.9046 (4)	0.0576 (10)	
H9	0.8171	0.1344	0.9725	0.069*	
C10	0.8893 (2)	0.1635 (3)	0.8859 (4)	0.0472 (8)	
H10	0.9310	0.1172	0.9419	0.057*	
N1	0.92249 (18)	0.3194 (2)	0.6043 (3)	0.0449 (7)	
N2	0.89867 (17)	0.2174 (2)	0.7915 (3)	0.0392 (6)	
O1	0.8926 (3)	−0.0960 (3)	0.7511 (4)	0.0902 (12)	
Cl1	0.92474 (5)	0.07552 (7)	0.56901 (9)	0.0448 (3)	
Co1	1.0000	0.20323 (5)	0.7500	0.0367 (3)	
O2	1.0000	−0.2593 (12)	0.7500	0.169 (6)	0.734 (17)
O3	1.0000	−0.4227	0.7500	0.174 (19)	0.269 (17)
H1A	0.911 (4)	−0.046 (3)	0.724 (5)	0.11 (2)*	
H1B	0.904 (4)	−0.085 (4)	0.836 (3)	0.11 (2)*	

### Atomic displacement parameters (Å^2^)

**Table:**

	*U*^11^	*U*^22^	*U*^33^	*U*^12^	*U*^13^	*U*^23^
C1	0.043 (2)	0.068 (3)	0.071 (3)	−0.0005 (18)	0.023 (2)	0.021 (2)
C2	0.061 (3)	0.076 (3)	0.083 (3)	0.003 (2)	0.027 (2)	0.042 (3)
C3	0.057 (3)	0.062 (3)	0.088 (3)	0.011 (2)	0.018 (2)	0.036 (2)
C4	0.046 (2)	0.050 (2)	0.070 (3)	0.0110 (17)	0.016 (2)	0.0131 (19)
C5	0.0330 (17)	0.0354 (17)	0.0441 (18)	−0.0011 (13)	0.0074 (14)	−0.0034 (14)
C6	0.0301 (17)	0.0368 (17)	0.0399 (17)	0.0027 (12)	0.0089 (14)	−0.0068 (14)
C7	0.0332 (19)	0.059 (2)	0.051 (2)	0.0130 (15)	0.0122 (16)	−0.0016 (17)
C8	0.045 (2)	0.077 (3)	0.064 (2)	0.014 (2)	0.032 (2)	0.001 (2)
C9	0.049 (2)	0.074 (3)	0.053 (2)	0.0112 (19)	0.0282 (19)	0.007 (2)
C10	0.0359 (18)	0.055 (2)	0.0461 (19)	0.0082 (16)	0.0174 (15)	0.0074 (17)
N1	0.0331 (15)	0.0416 (16)	0.0487 (16)	−0.0004 (12)	0.0120 (13)	0.0027 (13)
N2	0.0265 (14)	0.0427 (16)	0.0394 (14)	0.0044 (11)	0.0098 (11)	0.0005 (12)
O1	0.101 (3)	0.110 (3)	0.064 (2)	−0.057 (2)	0.044 (2)	−0.018 (2)
Cl1	0.0335 (5)	0.0513 (6)	0.0415 (5)	−0.0071 (3)	0.0128 (4)	−0.0062 (3)
Co1	0.0238 (4)	0.0402 (4)	0.0382 (4)	0.000	0.0097 (3)	0.000
O2	0.133 (10)	0.217 (14)	0.117 (8)	0.000	0.032 (7)	0.000
O3	0.20 (3)	0.28 (5)	0.11 (2)	0.000	0.12 (2)	0.000

### Geometric parameters (Å, °)

**Table:**

C1—N1	1.327 (5)	C8—C9	1.373 (6)
C1—C2	1.381 (6)	C8—H8	0.9300
C1—H1	0.9300	C9—C10	1.373 (5)
C2—C3	1.370 (7)	C9—H9	0.9300
C2—H2	0.9300	C10—N2	1.329 (5)
C3—C4	1.362 (6)	C10—H10	0.9300
C3—H3	0.9300	N1—Co1	2.151 (3)
C4—C5	1.379 (5)	N2—Co1	2.132 (3)
C4—H4	0.9300	O1—H1A	0.86 (6)
C5—N1	1.348 (4)	O1—H1B	0.85 (3)
C5—C6	1.476 (5)	Cl1—Co1	2.4298 (9)
C6—N2	1.351 (4)	Co1—N2^i^	2.132 (3)
C6—C7	1.378 (5)	Co1—N1^i^	2.151 (3)
C7—C8	1.377 (6)	Co1—Cl1^i^	2.4298 (9)
C7—H7	0.9300		
			
N1—C1—C2	122.8 (4)	C8—C9—H9	120.6
N1—C1—H1	118.6	N2—C10—C9	123.1 (3)
C2—C1—H1	118.6	N2—C10—H10	118.4
C3—C2—C1	118.2 (4)	C9—C10—H10	118.4
C3—C2—H2	120.9	C1—N1—C5	118.7 (3)
C1—C2—H2	120.9	C1—N1—Co1	125.8 (3)
C4—C3—C2	119.5 (4)	C5—N1—Co1	115.4 (2)
C4—C3—H3	120.2	C10—N2—C6	118.2 (3)
C2—C3—H3	120.2	C10—N2—Co1	125.5 (2)
C3—C4—C5	119.8 (4)	C6—N2—Co1	116.2 (2)
C3—C4—H4	120.1	H1A—O1—H1B	109 (5)
C5—C4—H4	120.1	N2—Co1—N2^i^	169.92 (15)
N1—C5—C4	120.9 (4)	N2—Co1—N1^i^	96.14 (11)
N1—C5—C6	116.1 (3)	N2^i^—Co1—N1^i^	76.58 (11)
C4—C5—C6	123.0 (3)	N2—Co1—N1	76.58 (11)
N2—C6—C7	121.4 (3)	N2^i^—Co1—N1	96.14 (11)
N2—C6—C5	115.7 (3)	N1^i^—Co1—N1	89.19 (16)
C7—C6—C5	122.9 (3)	N2—Co1—Cl1	91.72 (8)
C8—C7—C6	119.5 (3)	N2^i^—Co1—Cl1	95.26 (8)
C8—C7—H7	120.2	N1^i^—Co1—Cl1	171.64 (8)
C6—C7—H7	120.2	N1—Co1—Cl1	89.88 (8)
C9—C8—C7	118.9 (3)	N2—Co1—Cl1^i^	95.26 (8)
C9—C8—H8	120.6	N2^i^—Co1—Cl1^i^	91.72 (8)
C7—C8—H8	120.6	N1^i^—Co1—Cl1^i^	89.88 (8)
C10—C9—C8	118.8 (4)	N1—Co1—Cl1^i^	171.64 (8)
C10—C9—H9	120.6	Cl1—Co1—Cl1^i^	92.22 (5)
			
N1—C1—C2—C3	−0.8 (8)	C7—C6—N2—C10	−0.9 (5)
C1—C2—C3—C4	−0.5 (8)	C5—C6—N2—C10	180.0 (3)
C2—C3—C4—C5	1.0 (8)	C7—C6—N2—Co1	176.4 (3)
C3—C4—C5—N1	−0.3 (6)	C5—C6—N2—Co1	−2.7 (4)
C3—C4—C5—C6	−180.0 (4)	C10—N2—Co1—N2^i^	−136.3 (3)
N1—C5—C6—N2	1.5 (4)	C6—N2—Co1—N2^i^	46.6 (2)
C4—C5—C6—N2	−178.8 (3)	C10—N2—Co1—N1^i^	−93.0 (3)
N1—C5—C6—C7	−177.5 (3)	C6—N2—Co1—N1^i^	89.9 (2)
C4—C5—C6—C7	2.2 (5)	C10—N2—Co1—N1	179.3 (3)
N2—C6—C7—C8	1.9 (5)	C6—N2—Co1—N1	2.2 (2)
C5—C6—C7—C8	−179.1 (3)	C10—N2—Co1—Cl1	89.8 (3)
C6—C7—C8—C9	−1.5 (6)	C6—N2—Co1—Cl1	−87.3 (2)
C7—C8—C9—C10	0.3 (7)	C10—N2—Co1—Cl1^i^	−2.6 (3)
C8—C9—C10—N2	0.7 (6)	C6—N2—Co1—Cl1^i^	−179.7 (2)
C2—C1—N1—C5	1.5 (6)	C1—N1—Co1—N2	−179.5 (3)
C2—C1—N1—Co1	179.7 (4)	C5—N1—Co1—N2	−1.3 (2)
C4—C5—N1—C1	−1.0 (5)	C1—N1—Co1—N2^i^	7.5 (3)
C6—C5—N1—C1	178.7 (3)	C5—N1—Co1—N2^i^	−174.3 (2)
C4—C5—N1—Co1	−179.3 (3)	C1—N1—Co1—N1^i^	83.9 (3)
C6—C5—N1—Co1	0.4 (4)	C5—N1—Co1—N1^i^	−97.9 (3)
C9—C10—N2—C6	−0.4 (5)	C1—N1—Co1—Cl1	−87.7 (3)
C9—C10—N2—Co1	−177.4 (3)	C5—N1—Co1—Cl1	90.5 (2)

Symmetry codes: (i) −*x*+2, *y*, −*z*+3/2.

### Hydrogen-bond geometry (Å, °)

**Table:**

*D*—H···*A*	*D*—H	H···*A*	*D*···*A*	*D*—H···*A*
O1—H1A···Cl1	0.86 (6)	2.43 (5)	3.250 (4)	160 (5)
O1—H1B···Cl1^ii^	0.85 (3)	2.37 (4)	3.218 (4)	172 (4)

Symmetry codes: (ii) *x*, −*y*, *z*+1/2.
